# Our Contemporaries

**Published:** 1913-11

**Authors:** 


### OUR CONTEMPORARIES

---

*The Dietetic and Hygienic Gazette* of September, reprints from our May issue, Dr. Julius Richter's article on House Operations.

---

*The Medical Economist,* the official organ of the Associated Physicians' Economic League, entered the journalistic field in August. It is a monthly magazine whose worthy object is sufficiently indicated by the title. The two numbers issued thus far contain a number of interesting articles, dealing both with the individual and the professional economic problems at present before us for solution. We are authorized to offer a club rate with this Journal of $2.50 a year.

---

The *University Bison* is the organ of all departments of the University of Buffalo. It is edited by the students and by an advisory committee of the faculties. Seven numbers are issued each year. The subscription is $1.00. A club rate of $2.50 with the BUFFALO MEDICAL JOURNAL is offered.

